# Genomic architecture of resistance to latania scale (*H. lataniae*) in kiwifruit (*A. chinensis* var. *chinensis*)

**DOI:** 10.1186/s12870-023-04504-4

**Published:** 2023-10-31

**Authors:** Casey Flay, Jibran Tahir, Elena Hilario, Lena Fraser, Kate Stannard, Vaughan Symonds, Paul Datson

**Affiliations:** 1 Massey University, Palmerston North, New Zealand; 2 The New Zealand Institute for Plant and Food Research Limited, Auckland, New Zealand; 3 Kiwifruit Breeding Centre, Te Puke, New Zealand

**Keywords:** *Actinidia chinensis*, *Hemiberlesia lataniae*, Resistance, Scale, Kiwifruit, Breeding, Molecular

## Abstract

**Background:**

Latania scale (*Hemiberlesia lataniae* Signoret) is an armoured scale insect known to cause damage to kiwifruit plants and fruit, which ultimately reduces crop values and creates post-harvest export and quarantine issues. Resistance to *H. lataniae* does exist in some commercial cultivars of kiwifruit. However, some of the commercial cultivars bred in New Zealand have not inherited alleles for resistance to *H. lataniae* carried by their parents. To elucidate the architecture of resistance in the parents and develop molecular markers to assist breeding, these experiments analysed the inheritance of resistance to *H. lataniae* from families related to commercial cultivars.

**Results:**

The first experiment identified a 15.97 Mb genomic region of interest for resistance to *H. lataniae* in rtGBS data of 3.23 to 19.20 Mb on chromosome 10. A larger population was then QTL mapped, which confirmed the region of interest as the sole locus contributing to *H. lataniae* resistance. inDel markers mapping the region of low recombination under the QTL peak further narrowed the region associated with *H. lataniae* resistance to a 5.73 Mb region.

**Conclusions:**

The kiwifruit populations and genomic methods used in this study identify the same non-recombinant region of chromosome 10 which confers resistance of *A. chinensis* var. *chinensis* to *H. lataniae*. The markers developed to target the *H. lataniae* resistance loci will reduce the amount of costly and time-consuming phenotyping required for breeding *H. lataniae* scale resistance into new kiwifruit cultivars.

## Background

Kiwifruit are of significant commercial value to New Zealand, with exports alone earning NZD 2.7 billion by March 2022 which was a 2.4% increase compared to 2021 [1]. However, despite an ever-increasing overseas demand for kiwifruit and the associated financial gain, producing fruit of a quality suitable for export can be a difficult task. One of the obstacles to producing export grade kiwifruit is the occurrence of pests in the field, particularly the latania scale insect (*Hemiberlesia lataniae* Signoret) [2]. But a greater financial impact comes from exports to the most lucrative kiwifruit markets of Japan and China which list *H. lataniae* as quarantine restriction. Fruit exported to markets with quarantine restrictions need to be inspected individually to be free of *H. lataniae* at a high labour and packing throughput cost, making it the most costly insect to kiwifruit production in New Zealand. *H. lataniae* infests New Zealand’s two main commercially produced cultivars: the green-fleshed ‘Hayward’ and the gold-fleshed ‘Zesy002’, commonly known as Gold3, with fruit marketed as ZESPRI^tm^ SunGold. *H. lataniae* attacks the leaves, trunk, canes, and fruit of these cultivars [2]. In New Zealand, *H. lataniae* adults are all parthenogenic females that remain permanently fixed in the location established by mobile first instar crawlers [3]. They reach a reproductive age at 6–10 weeks and produce 50–100 crawlers over their lifetime of up to 36 weeks [3]. The crawlers hatch under their parent’s armoured scale or cap and migrate by crawling a short distance or being blown to new sites in the wind [4, 5]. Crawlers search for a feeding site for up to 48 h (personal observation) before inserting their stylet into the plant. Then they secrete a hard waxy cap to cover themselves, becoming sessile for the remainder of their lives [3, 6–8]. Conventional control with pest monitoring and pesticide application has proven somewhat effective, but a cultivar which is resistant to *H. lataniae* could save kiwifruit growers in New Zealand more than NZD 77 million per annum (personal communication Cathy McKenna).

Historically, New Zealand kiwifruit growers applied at least eight insecticide treatments per season to control *H. lataniae* on kiwifruit [9]. As a result of such heavy treatment loads, export rates became hindered as the fruit being produced was found consistently to carry pesticide residues at levels undesirable in foreign markets [10]. However, by 1997 the New Zealand kiwifruit industry had collectively agreed to move to integrated pest management under a scheme jointly developed by Plant and Food Research and ZESPRI®, named KiwiGreen®, which aimed to reduce pesticide residues within the fruit [10]. The KiwiGreen® system is still successfully in use in conventional orchards today, but some scale can remain on fruit post-harvest. This causes major delays in packing fruit as export regulations require that all insects be accurately identified before they are sold to overseas markets. The most sustainable, economically practical, and environmentally friendly approach to preventing *H. lataniae* attack has been identified as prevention rather than cure; that is, to breed kiwifruit cultivars resistant to insect attack thereby eliminating the issues associated with insect presence. This approach is particularly suited to organic growers who do not have the same treatment options available to them as conventional growers.

Like in other species, there is variation in resistance to armoured scale insects within kiwifruit species [11–15]. MG Hill, NA Mauchline, MK Jones and PW Sutherland [3] found that individuals resistant to *H. lataniae* will allow crawlers to spin a white cap over themselves in the first few days after settling, but insects on resistant plants do not develop a cap over 0.6 mm in diameter and do not develop into mature adults. Conversely, susceptible plants allowed unrestricted growth of *H. lataniae*, with scale caps growing to over 0.6 mm in diameter [3, 7, 15, 16]. Susceptible plants also allow the development of *H. lataniae* into reproductive adults if left to grow for more than 10 weeks [16]. When scale insects settle on the bark of canes of ‘Hort16A’, a wound periderm is formed under each scale insect. The wound periderm develops smaller cells with thicker cell walls and increased phenols than cane bark with no *H. lataniae* present. This was proposed to physically restrict the extension of the insect’s stylet into their feeding sites of parenchyma tissue and result in the resistance of ‘Hort16A’ to *H. lataniae* [3, 6, 17]. For an *Actinidia* population at Plant and Food Research, Te Puke, New Zealand, MG Hill, NA Mauchline, CH Cheng and PG Connolly [2] showed a “moderate heritability” of a hypersensitive response to *H. lataniae* in the cultivar ‘Hort16A’. However, due to the high susceptibility of ‘Hort16A’ to the bacterial pathogen *Pseudomonas syringae* pv. *actinidiae* (Psa), the ‘Hort16A’ cultivar has now been almost completely replaced by the cultivar ‘Zesy002’ in New Zealand, which, despite including ‘Hort16A’ in its pedigree, is susceptible to *H. lataniae* (personal communication: Nicola Mauchline).

Little is known about the molecular architecture governing the gene(s) responsible for the hypersensitive resistance response observed by MG Hill, NA Mauchline, MK Jones and PW Sutherland [3]. Effectors/elicitors of the hypersensitive response have been identified by RNA regulation in response to *H. lataniae* [6, 18]. However, the identification of hypersensitive response strongly suggests a gene-for-gene relationship between the plant’s dominant resistance gene and the insect’s dominant avirulence gene, as this is consistent across most species of higher plants that have been studied [19].

The two main commercial kiwifruit cultivars grown in New Zealand are susceptible to *H. lataniae*. This is due to two factors, firstly, *H. lataniae* resistance is viewed as a secondary trait for breeding purposes behind traits such as fruit flavour, yield, storage, and Psa resistance. Secondly, most elite gold breeding populations that possess these characteristics are tetraploid individuals, and resistance to *H. lataniae* has only been found in diploid gold fleshed populations. Moreover, phenotyping for resistance to *H. lataniae* is a costly and time-consuming process restricting funding for the large populations required to use unreduced gametes from Hort16A to integrate resistance into tetraploid populations and screen for resistance. One of the ways to reduce the time and labour involved with identifying resistant kiwifruit individuals is to develop molecular markers associated with resistance. Markers for resistance to *H. lataniae* could be implemented in breeding programs alongside sex markers which are already routinely used in marker assisted selection to increase the number of fruit-bearing females in the field. This will enable the cost-effective integration of resistance to *H. lataniae* in commercial kiwifruit breeding programs.

 The work presented here aimed to characterize the genetic architecture of resistance to *H. lataniae* and develop insertion-deletion (InDel) markers associated with that resistance. This was achieved by first using a small F_1_ family of kiwifruit segregating for resistance to identify regions of interest. A larger F_1_ family segregating for resistance was then used for QTL mapping using GBS data. The same population was mapped with inDel markers to further narrow the region of interest. Populations and data sources are summarised in Table 1.

**Table 1:**
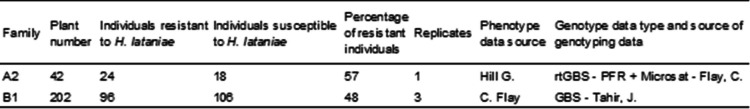
Kiwifruit family population sizes and results of phenotyping and genotyping for resistance to H. lataniae. These two families show an equal segregation of resistant and susceptible F1 seedlings

## Results

### Phenotyping the A2 and B1 families for *H. lataniae* resistance

Phenotyping the 18 plants from the A2 family showed an even 1:1 segregation ratio between resistance and susceptible individuals with nine individuals of each phenotype. The phenotype expression was consistent with prior research [15]. Phenotyping the parents of the A2 family confirmed resistance was coming from the Male 6 parent and susceptibility from the Female 5 parent. Phenotyping the larger B1 family, resulted in the identification of 96 resistant and 106 susceptible plants. This was consistent with the 1:1 segregation pattern seen in the A2 and A1 families, indicating a single dominant gene for resistance. The resistance phenotype observed in parents Female 6 and Male 6, along with the resistant individuals from the B1 family, was the same as that observed by MG Hill, NA Mauchline, MK Jones and PW Sutherland [3]. Crawlers spun a white cap over themselves in the first few days after settling, but they did not develop a cap over 0.8 mm in diameter and did not develop into mature adults. Susceptible plants allowed unrestricted growth of *H. lataniae* to adulthood over the assay duration, with scale caps over 0.8 mm in diameter. This was consistent with the phenotype observed in the susceptible control ‘Hayward’ used in the B1 family.

### Identifying genomic region of interest for *H. lataniae* resistance

To determine the genomic architecture of kiwifruit resistance to *H. lataniae*, pre-available rtGBS genotypes and *H. lataniae* resistance phenotype data for the A2 family were interrogated. The 32,352 variants from the re-analysed rtGBS data included 3800 variants heterozygous in parent samples which were sequenced with the family. These variants were analysed using a Kruskal-Wallis test which found 20 variants associated with the phenotype (*p* = 0.001) (Table 2).

**Table 2:**
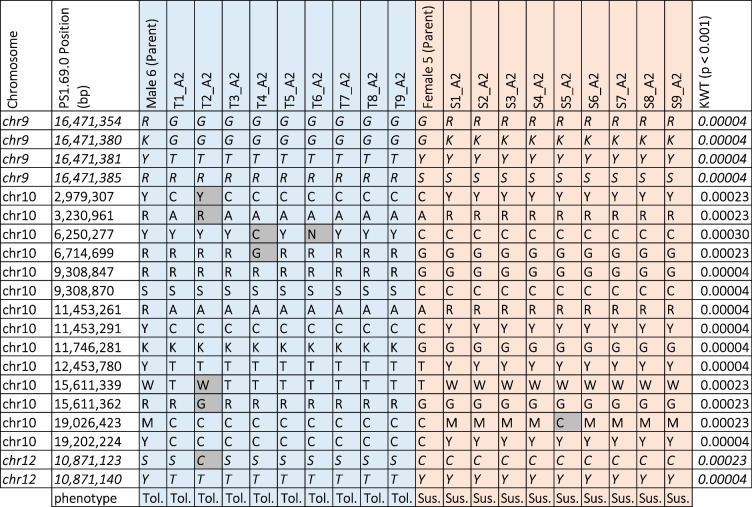
Alleles from the A2 family of kiwifruit with significant association (*p* = 0.001) between genotype and phenotype. The A2 family was an F1 family from a cross between resistant Male 6 and susceptible Female 5 which was sequenced with rtGBS. The bases that had significant association with phenotype were from the parent Male 6. Individuals resistant to *H. lataniae *are highlighted with blue fill, and susceptible individuals are highlighted by salmon fill. Variants not aligned with other individuals of their phenotype are highlighted in grey. Potentially misaligned reads were shown in italicised text. Variant bases were coded following IUPAC guidelines

All significant associations between markers and phenotype had a heterozygous male parent, which confirms the male as the contributor of resistance to *H. lataniae* in this family. The greatest number of markers was centred on chromosome 10, with fourteen markers showing a significant association with the phenotype (Table 2). Six markers were aligned to chromosomes 9 and 12, but as the marker positions on chromosomes 9 and 12 were less than 31 bp apart, it was assumed that each came from a single sequence read and were misaligned in reference PS1.69.0. Marker positions on chromosome 10 were found to segregate with resistance in a 15.97 Mb region of 3.23 to 19.20 Mb (Table 2). The individual T2_A2 was thought to be recombinant as a single arm of the chromosome was affected and the markers from the affected arm matched those of the susceptible individual markers from the Female 5 parent instead of those of other resistant individuals.

### QTL mapping the B1 family

**Fig. 1:**
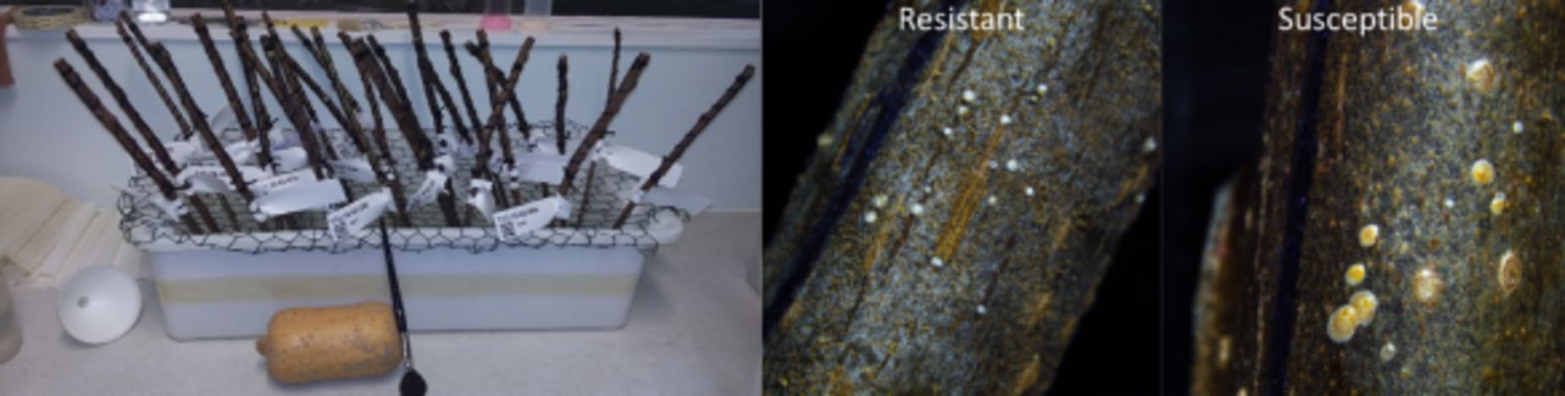
Woody cane bioassay to phenotype kiwifruit for *H. lataniae* resistance. *H. lataniae* crawlers are applied from a butternut squash (*Cucurbita moschata*) left. A 400-mm length of winter dormant kiwifruit cane is placed upright in water with wool wrapped around each cane. A section of cane resistant to *H. lataniae* is shown centre with no scale caps above 0.4 mm, and a cane susceptible to *H. lataniae* (right) with scale caps greater than 1.4 mm in diameter

To develop more precise markers for resistance, the distance between the gene and the marker needs to be as small as possible to avoid recombination between the gene and the marker. QTL analysis of the larger B1 family was proposed to provide more recombinant individuals to narrow down this association. QTL analysis of the provided GBS data showed association with the resistance phenotype on a 16 Mb section of chromosome 10 with a LOD score of over 6.4 at the tails of the peak, and 82.9 at the peak (Fig. 1). This peak explained 87.2% of the phenotypic variance. The QTL location aligns with previous variant data obtained from Plant and Food Research. The QTL analysis was re-run using chromosome 10 as a co-factor. No other peaks were observed that were above the threshold of significance at a LOD of 6.4. This indicated that one or multiple loci are located under the QTL peak, with no other sites contributing to resistance. However, due to the noise associated with the GBS data, it was difficult to assess which GBS markers were on either side of the of a recombination event in recombinant individuals. An indicator of error is the difference between markers on the same read in the new map. As two markers that were 87 bp apart were likely to be from the same read but were mapped to be 2.8 cM apart from each other. Recombination between markers so close together is unlikely.

The raw GBS sequencing files were mapped with (BWA-MEM) by the author to the reference genome known as PS1.69.0 [20]. QTL analysis of the GBS data showed 46 markers with LOD scores over the permutation test interval of 3.3 (Fig. 1). The marker with the highest LOD score of 105.5 explained 93.8% of the phenotypic variance. The markers with the highest LOD score landed in similar positions to those on the rtGBS analysis on chromosome 10. The increased marker density reduced some of the error, producing a sharper peak on the QTL map, but the error from the GBS data remained, restricting the ordering of markers at a fine level. To visualise the QTL region, LOD maps were made to look at the phenotype quantitatively with the 1–3 scale, but this did not fit the binary nature of resistance conferred by Female 6. To test whether this had an effect, a Kruskal-Wallis test was run on the GBS data in Map QTL. The results confirmed the significant association of markers on chromosome 10 with the phenotype.

### Markers for *H. lataniae* resistance

To develop markers for the B1 family in the region under the QTL peak on chromosome 10, 22 inDel markers were designed, which spanned a region of 4.19 to 13.85 Mb on chromosome 10 (Table 3). Because 15 plants were found to recombine between the resistance markers, the markers could be used to map association with resistance. The region of interest was narrowed to a 5.73 Mb region of 5.17 to 10.90 Mb. At this resolution, only three recombinant plants were found, restricting further mapping resolution. The region associated with *H. lataniae* resistance was viewed in JBrowse [21]. This region was found to contain 139 manually annotated genes with a low recombination rate, likely caused by the presence of the centromere in this region [22].

**Table 3:**
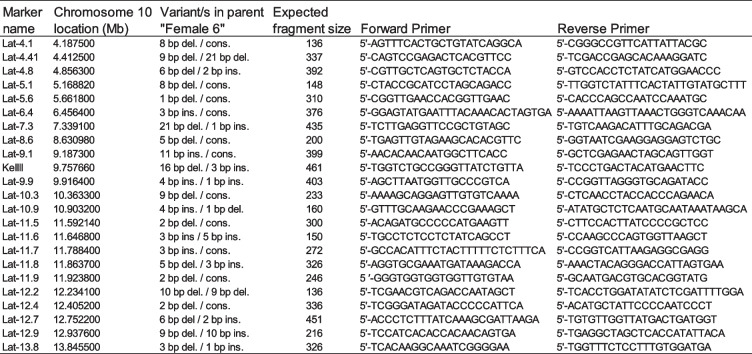
InDel markers used to map recombinant individuals from the B1 family of kiwifruit. All markers were designed to have a size between 136 and 461 bp of 4.18 Mb to 13.85 Mb along chromosome 10. All primers used a melting temperature of 58.5 – 60.5 °C. All forward primers had a TGTAAAACGACGGCCAGT M13 tag added onto the 5’ end. Variants present in the parent Female 6 are shown with both alleles separated by a forward slash. ‘Ins.’ refer to insertions, and ‘del.’ deletions present in the amplified fragment, while ‘cons.’ indicates consensus with reference

## Discussion

The main commercial kiwifruit cultivars grown in New Zealand are susceptible to the armoured scale insect Latania scale - *H. lataniae -* (personal communication Cathy McKenna). This is primarily due to the high cost and slow turnaround of breeding kiwifruit from seed to a vine that can be reliably phenotyped for resistance to *H. lataniae*. However, these restrictions on conventional phenotyping for kiwifruit breeding can potentially be overcome using molecular markers developed to differentiate resistant and susceptible individuals at the seedling stage for the selection of parents resistant to *H. lataniae*. But before markers can be developed, the genetic architecture of resistance or gene/s markers associated with the trait needs to be identified. Markers developed with close association to resistance loci can provide a cost-effective solution for commercial breeding programs to incorporate resistance to *H. lataniae* into new kiwifruit cultivars. Ideally, cultivars that incorporate resistance to *H. lataniae* should not need control of this insect.

**Fig. 2:**
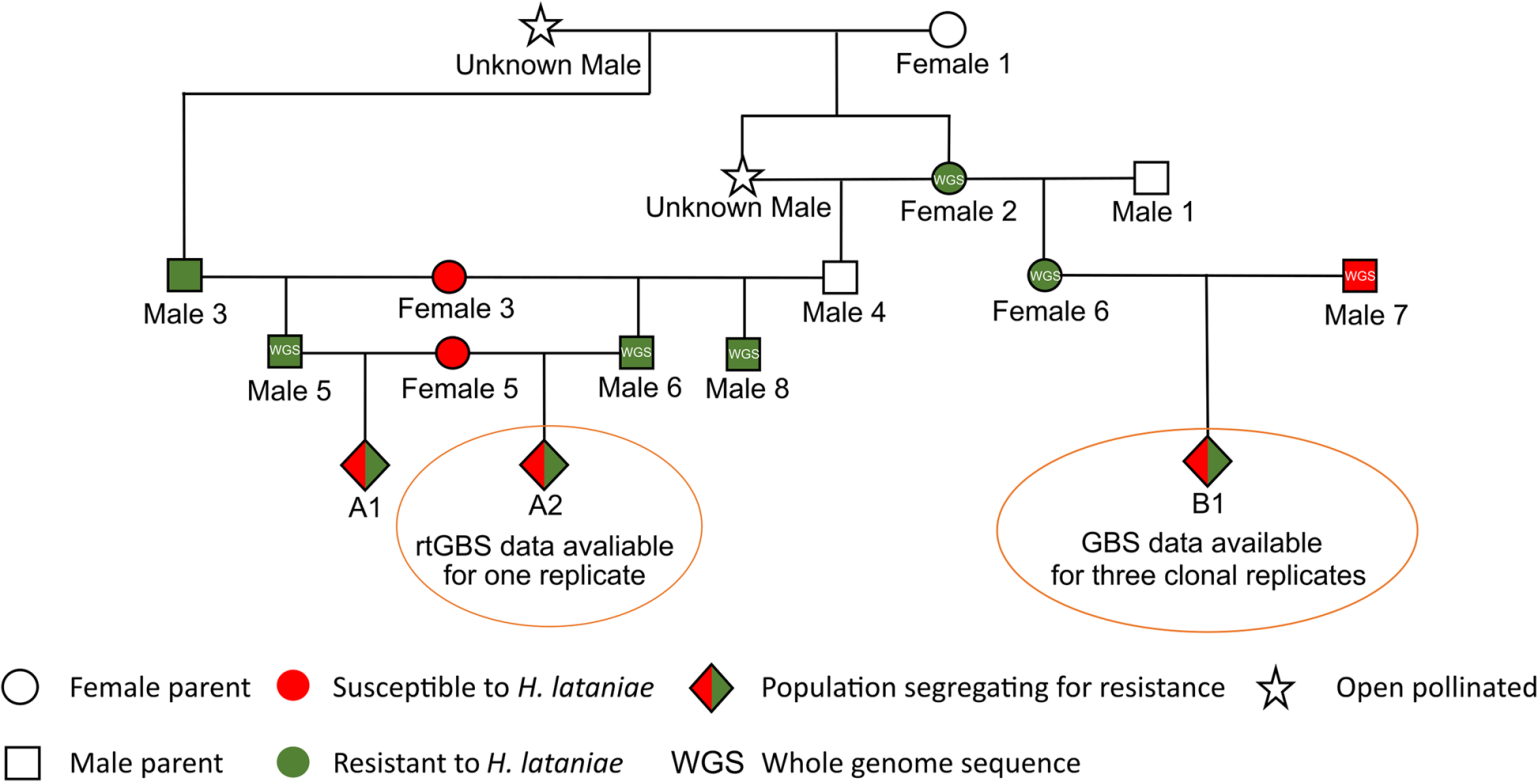
Pedigree of the A2 and B1 kiwifruit families. The circle nodes indicate females, with the square nodes as males. Red indicates susceptible with green resistant to *H. lataniae*. Diamond nodes indicate families with the number of genotypes in the family. The A2 and B1 families having 18 and 202 individuals respectively. A star node in the pedigree tree indicates that pollen was from an open cross thus, the male parent is unknown. Inheritance of resistance to *H. lataniae *in the A2 and B1 families was likely from one of the parents from the first recorded cross of an unknown male to Female 1. From this cross, the resistance allele passed onto the resistant Female 2 and Male 3. Female 2 likely passed the resistance allele onto Female 6 and Male 4, then Male 4 passed resistance to Male 6. Male 3 may have passed the same resistance inherited from Female 1 onto Male 5 resulting in the related A1 family segregation

To identify the genetic architecture of resistance to *H. lataniae* in kiwifruit (*A. chinensis*), two families descended from a common ancestor (Fig. 2) were studied. From the initial rtGBS and phenotype data analysed from A2 family a 15.97 Mb region on chromosome 10 was identified as the sole contributor to *H. lataniae* resistance. To verify this association a larger related “B1” family was QTL mapped. The QTL map confirmed the association on chromosome 10. However, the QTL map showed an unusually high LOD score. This may have been due to a high sampling error or DNA contamination in the GBS markers, and possibly phenotyping error. These issues also inhibited the accurate ordering of the GBS markers for the B1 population. To further utilise the population, molecular markers were designed to target the region under the QTL peak associated with resistance. This work narrowed the region associated with resistance to a locus spanning a 5.73 Mb region on chromosome 10 of 5.17 to 10.90 Mb. The ability of the inDel mapping approach to narrow down the region of interest further was limited by the lack of individuals with recombination in the region. This was likely due to a centromere being present in the region of interest reducing the rate of recombination around its binding site [23, 24]. The identification of a single locus which triggers a hypersensitive response to *H. lataniae* [3] is consistent with the gene-for-gene interaction typically observed in plants that have a prolonged intimate relationship with pests such as Hessian fly larvae [25], nematodes [26], or aphids [19, 27–30]. Since *H. lataniae* is immobile once feeding, has a lifelong relationship with its host, and is a parthenogenic insect in New Zealand, it fits with the hypothesis that Female 6 has a dominant resistance gene which responds to the dominant avirulence gene in *H. lataniae* by stimulating the salicylic acid signalling cascade found by MG Hill, NA Mauchline, MK Jones and PW Sutherland [3].

As well as identifying a genetic basis for resistance to *H. lataniae* in kiwifruit, this study also identified several InDel markers associated with resistance that can be used for selective breeding in kiwifruit. Molecular markers have been employed in kiwifruit science to develop linkage maps [20, 31, 32], characterise kiwifruit diversity [27, 33], and to make associations with Psa resistance [34]. However, no pest or disease resistance markers are used in the green, gold and red kiwifruit breeding programs (personal communication Alan Seal) [35]. The inDel markers developed for this study are the first published molecular markers for resistance to *H. lataniae* in kiwifruit and, to our knowledge, are the first molecular markers for resistance to armoured scale insects in plants. These markers have been used to select for *H. lataniae* resistance in five populations that use Female 6 as a parent (unpublished research).

The bioassay method developed for this study reduced handling error by leaving plants in the glasshouse when *H. lataniae* insects were applied to canes. This was possible due to the higher humidity generated by the wet tissue paper applied around the site of application. It is thought that this allowed greater numbers of crawlers to settle by preventing the thin flat crawlers from becoming dehydrated before finding a suitable site to settle. The modified bioassay method reduced the workload and time taken for the assay setup compared to the method described by MG Hill, KV Wurms, MW Davy, E Gould, A Allan, NA Mauchline, Z Luo, AA Chee, K Stannard and RD Storey [6] for live plant assays, which involved seeding plants in the lab and moving plants to the glasshouse after settlement. This is likely to have reduced environmental error within treatments which could have been introduced through environmental variation between the lab and the glasshouse, as well as reducing damage to insects and plants due to handling. The use of three clonal replicates was a great advantage, allowing individuals with incorrect phenotyping results in one of the three plant clones to be identified and removed from the analysis. Despite the measures to reduce phenotyping error using the clonal replicate data, errors were detected through investigation of markers in recombinant individuals in the inDel based map of the B1 family. The erroneous susceptible phenotype calls had markers for resistance which did not fit with other recombinant susceptible individuals or other recombinant resistant individuals. If there were insufficient recombinant individuals to check whether the region of interest of the incorrect call lined up with the rest, an incorrect region of interest position could have been established. This was a particularly important issue when narrowing down the region associated with resistance as the low number of recombinant individuals reduced the number of consistent boundary calls to increase confidence and correct errors such as these. Yet with a less distinctive and a poorly characterised phenotype, the reassessment of phenotyping calls to confirm phenotyping error may not have been possible.

## Conclusions

Using the markers developed in these experiments for prediction of *H. lataniae* resistance should assist plant breeding, but it is important to note that the resistance observed in the A2 and B1 families was inherited from the common resistant ancestors of Female 2 and Male 3 (Fig. 2). Testing these markers on unrelated *Actinidia* populations that are resistant to *H. lataniae* may establish whether resistance to *H. lataniae* is conferred by a single gene across *Actinidia* germplasm or whether there are many variants of resistance to *H. lataniae* in *Actinidia* species. However, because the 5.73 Mb region is large, the *H. lataniae* resistance loci can dissociate from the markers designed to target it. This is particularly a problem when testing markers on unrelated populations which share the resistance gene, as there is a greater chance that linkage between the marker and resistance gene has been broken. The large region associated with resistance is also an issue when using these markers in future breeding programs as the parents of populations will still need to be phenotyped to make sure that the association between the marker and resistance gene has not been broken. If the marker association is intact these markers could be used to screen seedlings for individuals resistant to *H. lataniae*.

Using the inDel markers developed here for prediction of *H. lataniae* resistance should significantly reduce the cost, time, workload and facilities required for *H. lataniae* phenotyping. These markers would be particularly useful for developing male polleniser cultivars with resistance to *H. lataniae*, since there are fewer competing traits in pollenisers other than pollen quantity and performance. Because pollenisers are often planted in blocks with fruiting plants, future pollinisers with resistance to *H. lataniae* would prevent these individuals from spreading *H. lataniae* crawlers to fruiting plants. A commercial polliniser could be crossed to an individual descending from the resistant ancestor of Female 2 and Male 3. Then, the resistance markers developed here could be used to select resistant seedlings.

While a lack of recombination in the 5.73 Mb region was an impediment for gene identification, it is an advantage for using markers in plant breeding programs. This is because markers within regions of low recombination have a lower risk of being disassociated from their target gene by recombination and thus, will provide fewer false positives and false negatives to marker calls. Moreover, the non-recombinant region is a great target for breeding resistance to *H. lataniae* into kiwifruit as the parthenogenic nature of *H. lataniae* in New Zealand may not allow the adaptation of avirulence genes in *H. lataniae* as rapidly as in those species which undergo sexual recombination. Further, the selection pressure to overcome the resistance gene in a polyphagous species such as *H. lataniae* will be lower than that in a monophagous species dependent for its survival on a specific cultivar. Plants which carry this resistance could be a great advantage to future kiwifruit cultivars. Future work should investigate whether other genes for resistance exist in families unrelated to Female 1.

## Materials and methods

### Plant material

The B1 family of 202 individuals came from the *H. lataniae* resistant female parent (Female 6) and the susceptible male parent (Male 7) (Fig. 2) to make an F1 cross. The seedlings making up the population were cloned, by leaf tissue culture at Plant and Food Research by J Tahir, S Gardiner, H Bassett, D Chagné, C Deng and L Gea [36], to make three replicates per genotype. Two resistant clonal plants of Female 6 and two susceptible plants of the cultivar ‘Hayward’ were included as controls with each replicate. Plants were grown up to 1.5 m high in 2.8-L planter bags in a plastic house in Palmerston North, New Zealand. Yates Thrive all-purpose liquid plant food, supplied by Mitre 10 (Te Puke, New Zealand), was applied at 3-month intervals at the recommended dose. At a minimum of eight weeks before phenotyping, each clonal replicate was transported to the environment where the experiment would be performed. All plants were re-potted into 4.5-L pots, with potting mix and Osmocote-Exact-Protect slow-release fertiliser tablets, both provided by Daltons (Mount Manganui, New Zealand). One replicate was grown in an open walled glasshouse at Plant and Food Research, Palmerston North New Zealand with an average temperature over the 10 weeks assessment of 22.5 ± 4 °C. The other two replicates were relocated to Plant and Food Research, Te Puke, New Zealand. One of these replicates was grown in a fan vented glasshouse at 20 ± 7 °C, while the other was in a shaded passively vented plastic house at 19 ± 11 °C. These locations were selected to give a measure of the environmental influence on the resistant phenotype.

### DNA material

DNA extraction methods for the pre-genotyped A2 family, and the pre-genotyped B1 families can be found in [37] and [36] respectively. DNA for marker development from the B1 family was extracted from glasshouse grown plants. Young actively growing leaves < 40 mm in length were collected and ground with a pestle and mortar in liquid nitrogen. DNA was extracted using a Qiagen DNeasy® Plant Mini Kit, following the manufacturer’s protocol [38]. DNA quality was analysed on a NanoDrop® 1000 spectrophotometer (Thermo Fisher Scientific) for the 260/280 absorbance ratio and DNA quantity. Samples with A260/A280 ratios outside the 1.6–2.2 range were re-extracted [39].

### Insect material

To allow cut cane and live plant bioassays to be implemented when required, a colony of *H. lataniae* was maintained in a sealed room at 20 ± 2 °C and 55 ± 5% RH at the Te Puke lab at Plant and Food Research, New Zealand. The colony of *H. lataniae* was grown on fruit of butternut squash (*Cucurbita moschata*). Crawlers from squash infested with adult *H. lataniae* were brushed onto clean undamaged squash every second day. Population brushing was done between 8 am and 11.00 am to coincide with the peak in crawler emergence from under adult scale caps (personal observation, Kate Stannard). It was critical to clean the squash thoroughly, as population contaminants of *Aspidiotus nerii* or *Hemiberlesia rapax* can be introduced which are difficult to distinguish from *H. lataniae.* Net pantyhose was applied around uninfested squash before brushing to assist scale establishment. The pantyhose was removed after 14 days. Squash were kept in bins with a mesh vented lid that was sealed closed with double sided tape between the bin’s lid and base to prevent predation from earwigs (*Forficula auricularia*) and parasitoid wasps of *Encarsia* spp.

### Phenotyping for *H. lataniae* resistance in the A2 family using the cut cane method

**Fig. 3:**
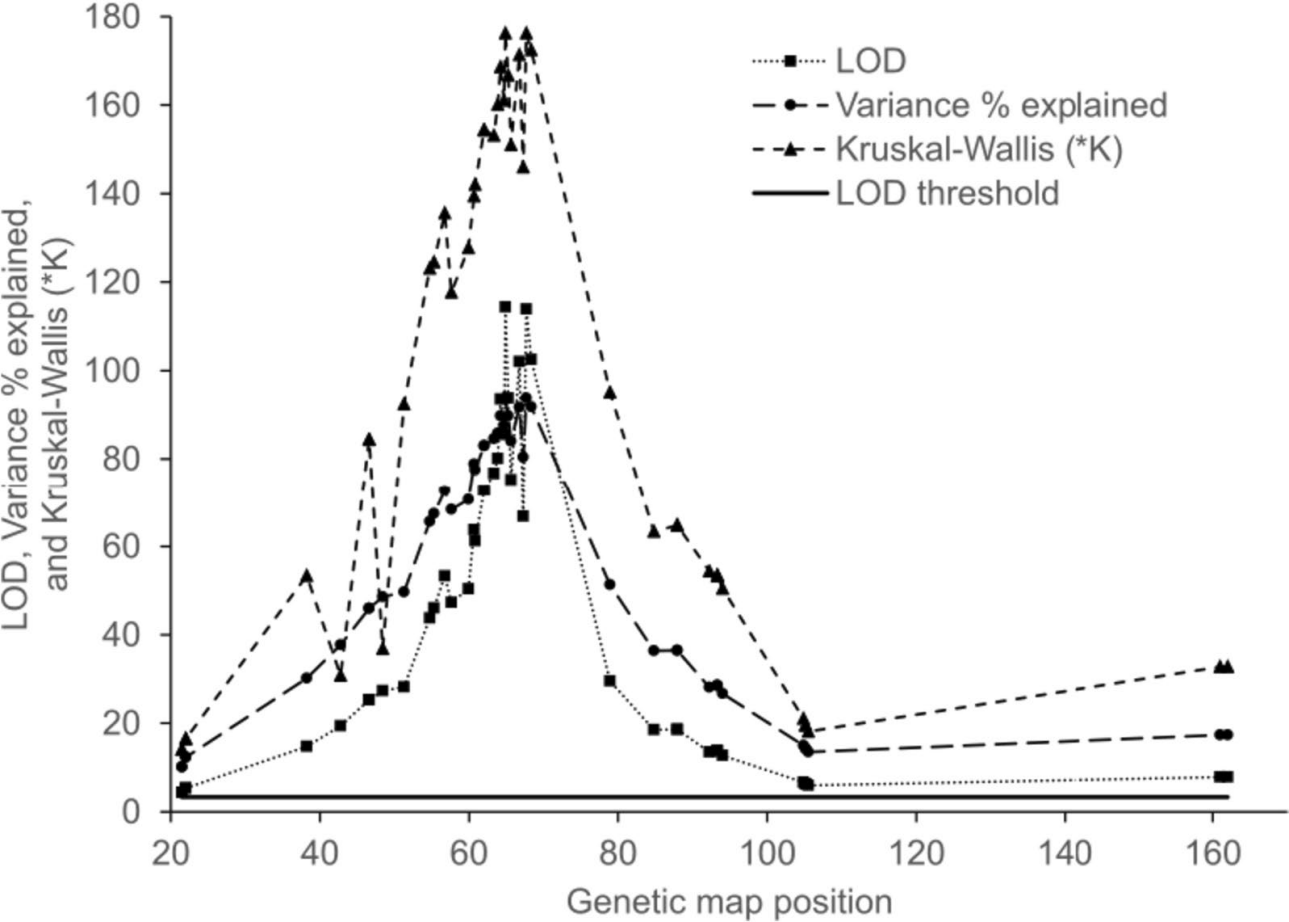
QTL map of the 20 Mb length of chromosome 10 from the B1 kiwifruit family. The genetic map shows a cluster of robust markers landing between 60 and 80 cM with LOD scores in excess of 100. The marker with the highest LOD score accounted for 93.8% of phenotypic variance. Kruskal-Wallis test values agreed with the LOD score (*p*=0.001)

Two methods were used to establish the *H. lataniae* resistance phenotype of kiwifruit in this study: a cut cane method was developed by MG Hill, NA Mauchline, CH Cheng and PG Connolly [2] to phenotype the A2 family, and a similar live plant method used to phenotype the B1 family. The cut cane method used two 1.2-m sections of one-year-old kiwifruit cane, at least 15 mm thick. Cane wood was taken from field grown plants in the Te Puke Plant and Food Research Centre orchard. These were placed into long plastic bags, sealed and moved to cool storage until the bioassay could be completed. The order of genotypes was randomised. Three 400-mm lengths of the healthiest canes greater than 15 mm in diameter were selected for the bioassay, with the aim of making three replicates for each genotype. Lateral buds were removed as close to the cane as possible. Wax was applied to cut areas, excluding the basal end of the cut section, and wool was wound around a 150-200-mm Sect. 20 mm down from the apical end of the cane. The canes were then labelled and placed horizontally side by side on a tray. *H. lataniae* crawlers were brushed onto the canes from a population of adult scale (Fig. 3). Canes were carefully lifted and the basal end placed in containers with 20 mm of water. Canes were kept upright standing them through wire mesh over the top of the bin holding the containers (Fig. 3). After ten days the wool was removed and the scale insects were left to develop for ten weeks in a sealed room at 20.5 ± 1.3 °C and 51.5 ± 7% relative humidity. After the scale development period they were measured using the same sizing and calling parameters as in the potted plant bioassay.

### Phenotyping for *H. lataniae* resistance in the larger B1 family using the live plant method

The cut cane method of phenotyping was effective for small numbers of individuals when sufficient *H. lataniae* scale crawlers were available. However, the large size of the B2 family and the small canes of some plants made them unsuitable for the cut cane bioassay. A higher throughput method was required to complete the phenotyping of all replicates before winter, when the growth of *H. lataniae* slows. Therefore, the live plant method described in MG Hill, KV Wurms, MW Davy, E Gould, A Allan, NA Mauchline, Z Luo, AA Chee, K Stannard and RD Storey [6] was modified to increase the bioassay throughput for the three replicates of 202 individuals in the B1 family. The adapted assay method used live plants with crawlers applied to plants in the glasshouse instead of cut- canes in the lab. For the bioassay, plants were prepared by wrapping wool around a 20-cm section of kiwifruit cane around 20 cm above the soil level. Scale crawlers were prepared by brushing crawlers off the rearing colony into a sample container coated with Fluon® paint to prevent crawler escape. Crawler collection was done between the times of 9 am and 11.00 am to utilise the peak production of crawlers from the colony. The sample container with crawlers was transported to the glasshouse and around 150 crawlers transferred onto the wool wrapped sections of plant canes with a paint brush. A damp paper towel was wrapped around the wool wrapped sections where crawlers were applied to increase the humidity of the settlement site. Paper towels were kept wet for three days by spraying water onto paper towels twice a day. Three days after crawler application and crawler establishment was checked by inspecting the cane for the small white caps made by the sessile crawlers. Wool and paper towels were removed from plant canes with more than 10 visible scale caps. Plant canes with fewer than 10 visible caps had crawlers reapplied. At the completion of the 10-week period from scale crawler application, the size of each scale cap was recorded as into two size classes: a smaller scale category of less than 0.6 mm and larger scale category with scale caps greater than 0.6 mm. Plant canes with greater than five scale caps larger than 0.6 mm were recorded as ‘susceptible’. Plant canes with greater than ten scale in the smaller < 0.6 mm size range and no scale caps in the larger scale category were recorded as ‘resistant’. The smaller < 0.6 mm size range corresponded to the resistance present in the mother Female 6, restricting the growth of the scale to around 0.2 mm. The larger > 0.6 mm size range corresponded to the susceptibility of the father Male 7, allowing the growth of scale to around 1.2 mm. The first replicate had *H. lataniae* applied to plants in Te Puke in a fan-vented and mist-humidified glasshouse at 20.8 °C plus/minus standard deviation (±) 3.9 °C, and relative humidity (RH) of 72.7% ± 12.2% RH, over six days from 27 to 2018. The second replicate also had crawlers applied to plants in Te Puke in a separate passively-vented plastic house with environmental conditions of 17.7 °C ± 4.1 °C and 74 ± 13.2% RH over three days from 13 to 2018. The third replicate was phenotyped in a passively-vented glasshouse at Massey University Palmerston North, under conditions of 22.5 °C ± 3.8 °C and 61.8% ± 14.6% RH.

### Identifying genomic region of interest for *H. lataniae* resistance in the A2 family

The identification of loci for scale resistance began by analysing the available genotypic and phenotypic data of 18 diploid *A. chinensis* F1 individuals from the A2 family (Fig. 2) provided from a study done by C Cheng, R Crowhurst, E Hilario, P Datson, L Barron, K Manako, C Deng, N De Silva and M Bomert [37]. This population was utilised as it had been phenotyped for *H. lataniae* scale resistance based on a cut cane bioassay (described later in this paper) available (Plant and Food Research, Te Puke, New Zealand, unpublished data). There were also rtGBS genotype data available for the same individuals, as the A2 population had been previously used for a trial of genomic selection [37]. The A2 family also showed an even 1:1 segregation of resistant and susceptible phenotypes in the field grown F1 individuals. The A2 family was derived from a cross between a resistant parent, Male 6, and a susceptible parent, Female 5 (Fig. 2). The A2 family was especially interesting because a related but independent family was showing the same 1:1 segregation of resistant and susceptible phenotypes, indicating that a single locus underlies resistance. The related A1 validation family shared the susceptible mother (Female 5) with the A2 family, but was crossed with a different resistant father, Male 5. Male 5 is related to Male 6 by sharing a mother (Female 3) and having a related father (Fig. 2).

The A2 family had been rtGBS genotyped following the method of E Hilario, L Barron, CH Deng, PM Datson, N De Silva, MW Davy and RD Storey [40] and data consisting of 39,000 variants for each of the 10 resistant and 10 susceptible plants were made available for this study. Data in the form of International Union of Pure and Applied Chemistry (IUPAC) degenerate base symbols for each variant were imported into R statistical software for analysis. Variant calls were recoded to numeric for data checking, filtered to remove homozygous variants, and filtered to remove variants with bases the same as those of the resistant male parent [41]. A Kruskal-Wallis test [42] was run for each VARIANT site against each individual’s phenotype call in the remaining 3800 variants heterozygous in the resistant parent. The Kruskal-Wallis test evaluated the null hypothesis for association between phenotype and genotype at each VARIANT site. Variants with significant phenotypic association (p ≤ 0.001) were also checked manually to confirm they segregated as expected i.e. with a variant inherited from the resistant male parent being present in all the resistant individuals and absent in the susceptible individuals. It was expected that the Kruskal-Wallis test would identify a genomic region of interest for scale resistance as a series of linked markers from one region in the genome. Markers for this region could then be identified and tested in the larger B1 population to see if they continued to show association with scale resistance.

### QTL mapping the larger related B1 family

To build on the results from the A2 family and to validate the association between the 3.23 to 19.20 Mb region on chromosome 10 in the A2 family, a larger family related to the resistant parent of the A2 family was sought to map the QTL associated with resistance. A family of 202 GBS-genotyped individuals with three clonal replicates for each individual was made available from a population developed by J Tahir, S Gardiner, H Bassett, D Chagné, C Deng and L Gea [36]. This F1 cross was phenotyped for *H. lataniae* resistance or susceptibility using the live plant bioassay described later in this paper. All experimental replicates were combined after removing individuals that had inconsistent phenotype calls across replicates.

The GBS genotype data were collected by J Tahir, S Gardiner, H Bassett, D Chagné, C Deng and L Gea [36] and sequenced at the Australian Genome Research Facility (AGRF) on an Illumina HiSeq with 100 bp paired end reads. Data were initially made available from a linkage map in JoinMap format and phased with allele codes < llxlm>. the raw FASTA files from AGRF were aligned using a BWA-MEM/Samtools pipeline aligned to the Red5:PS1.69.0 reference genome available from NCBI [20]. The genotype data from the B1 family were used to make linkage maps in JoinMap® 4. Three plants were removed from the dataset while making the linkage map because about a third of the markers coded as null. Around a quarter of the markers were identical to others and were removed before calculating the linkage map. The linkage map data were exported as .map and LOC files for QTL mapping in MapQTL 5 [43]. A cross pollinators (CP) population type was chosen to match the heterogeneously heterozygous and homozygous diploid parents of the studied population. The interval mapping method was used to detect QTL between markers and phenotype [43]. The genome-wide significance level thresholds for interval mapping were estimated to be 3.3 when using a permutation test with 1000 replicates. This threshold was measured by comparing individual chromosome thresholds and selecting the highest threshold as the threshold for the entire genome and any LOD scores under 3.3 were considered insignificant. Because the phenotype data for this trait were binary, the Kruskal-Wallis method was also applied in MapQTL 6 [43].

### Genetic markers for *H. lataniae* resistance in the B1 family

To lower the risk of the association between resistance gene/s and marker/s being broken through recombination, selected markers should be as close to the resistance genes as possible. To achieve this, the genome was mapped with inDel markers covering the region of interest under the QTL peak. Along with the reference genome, binary alignment map (BAM) files of the B1 family parents and individuals resistant to *H. lataniae*, i.e. Female 2, Male 5, and Male 6, (Fig. 2), were loaded into an integrated genome viewer - IGV [44] as tracks to visually identify informative alleles and check marker quality. These resistant individuals were used as checks because of their likely inheritance of the resistance haplotype from a common ancestor as determined from their pedigree and to ensure the resistance alleles and null alleles selected were informative.

InDels over 4 bp in length that were in the resistant parent (Female 6), but not in the susceptible parent (Male 7) were checked to see if they were consistent with reads from the other resistant parents. Twenty-two InDel markers were selected to span the QTL region. Primers were generated to target informative InDels in Geneious with Tm ± 1.5°C, and product lengths between 136 and 461 Bp. Primers were generated using the *A. chinensis* reference genome scaffold ‘Red5’ [20]. Primer binding sites were checked in IGV to see if they had any InDels in Female 6, Male 7, Female 2, Male 5, or Male 6. Twenty-four markers were selected after filtering on the above criteria, landing at 4.1, 4.8, 5.1, 5.6, 6.4, 7.3, 8.1, 8.6, 8.8, 9.1, 9.9, 10.3, 10.9, 11.1, 11.5, 11.6, 11.7, 11.8, 11.9, 12.2, 12.4, 12.7, 12.9, and 13.8 Mb along chromosome 10. For mapping, PCR reactions were set up as per M Schuelke [45]. This involved tagging the forward primers with a dye labeled-M13(-21) primer tail at the 5’ end. PCR reagents and conditions were as presented in M Schuelke [45]. For a final 10-µl reaction volume in a Perkin-Elmer Standard PCR reaction buffer, eight pmol of reverse primer and dye labeled-M13(-21) primer, two pmol of the forward primer, 0.2 mM dNTPs, 50–100 ng template DNA, and 1 U Platinum Taq DNA polymerase were used. Conditions of the PCR amplification: 94 °C (5 min), then 30 cycles at 94 °C (30 s) / 56 °C (45 s) / 72 °C (45 s), 8 cycles 94 °C (30 s) / 53 °C (45 s) / 72 °C (45 s), final extension at 72 °C for 10 min. To assess fragment lengths, 1 µl of the PCR product was added to 22 µl formamide and 0.5 µl ROX standard (PerkinElmer) and run on an Applied Biosystems 3130 Genetic Analyzer.

## Data Availability

Data generated or analysed during the current study were included in the manuscript. The raw data is available from the corresponding author on reasonable request.
